# Sex-associated differences in mitochondrial function in human peripheral blood mononuclear cells (PBMCs) and brain

**DOI:** 10.1186/s13293-018-0193-7

**Published:** 2018-07-25

**Authors:** C. Silaidos, U. Pilatus, R. Grewal, S. Matura, B. Lienerth, J. Pantel, G. P. Eckert

**Affiliations:** 1 Nutrition in Prevention and Therapy, Institute for Nutritional Sciences, University of Giessen, Wilhelmstr. 20, 35392 Giessen, Germany; 2 Institute for Neuroradiology, Goethe University Frankfurt, Schleusenweg 2-16, 60528 Frankfurt/Main, Germany; 3 Institute of General Practice, Goethe University Frankfurt, Theodor-Stern-Kai 7, 60590 Frankfurt/Main, Germany; 4 Department of Psychiatry, Psychosomatic Medicine and Psychotherapy, University Hospital Frankfurt, Heinrich-Hoffmann Str. 10, 60528 Frankfurt/Main, Germany; 5 Brain Imaging Centre, Schleusenweg 2-16, 60528 Frankfurt/Main, Germany

**Keywords:** Sex differences, Blood cells, MR spectroscopy, Mitochondria, Mitochondrial respiration, *N*-Acetylaspartate

## Abstract

**Background:**

Alzheimer’s disease (AD) is the most common form of dementia, and it affects more women than men. Mitochondrial dysfunction (MD) plays a key role in AD, and it is detectable at an early stage of the degenerative process in peripheral tissues, such as peripheral mononuclear blood cells (PBMCs). However, whether these changes are also reflected in cerebral energy metabolism and whether sex-specific differences in mitochondrial function occur are not clear. Therefore, we estimated the correlation between mitochondrial function in PBMCs and brain energy metabolites and examined sex-specific differences in healthy participants to elucidate these issues.

**Methods:**

The current pilot study included 9 male and 15 female healthy adults (mean age 30.8 ± 7.1 years). Respiration and activity of mitochondrial respiratory complexes were measured using a Clarke-electrode (Oxygraph-2k system), and adenosine triphosphate (ATP) levels were determined using a bioluminescence-based assay in isolated PBMCs. Citrate synthase activity as a mitochondrial marker was measured using a photometric assay. Concentrations of brain energy metabolites were quantified in the same individuals using ^1^H-magnetic resonance spectroscopy (MRS).

**Results:**

We detected sex-associated differences in mitochondrial function. Mitochondrial complexes I, I+II, and IV and uncoupled respiration and electron transport system (ETS) capacity in PBMCs isolated from blood samples of females were significantly (*p* < 0.05; *p* < 0.01) higher compared to males. ATP levels in the PBMCs of female participants were approximately 10% higher compared to males. Citrate synthase (CS) activity, a marker of mitochondrial content, was significantly (*p* < 0.05) higher in females compared to males. Sex-associated differences were also found for brain metabolites. The *N*-acetylaspartate (NAA) concentration was significantly higher in female participants compared to males in targeted regions. This difference was observed in white matter (WM) and an area with a high percentage (> 50%) of gray matter (GM) (*p* < 0.05; *p* < 0.01). The effect sizes indicated a strong influence of sex on these parameters. Sex-associated differences were found in PBMCs and brain, but the determined parameters were not significantly correlated.

**Conclusions:**

Our study revealed sex-associated differences in mitochondrial function in healthy participants. The underlying mechanisms must be elucidated in more detail, but our study suggests that mitochondrial function in PBMCs is a feasible surrogate marker to detect differences in mitochondrial function and energy metabolism in humans and it underscores the necessity of sex-specific approaches in therapies that target mitochondrial dysfunction.

**Electronic supplementary material:**

The online version of this article (10.1186/s13293-018-0193-7) contains supplementary material, which is available to authorized users.

## Background

Mitochondria are maternally inherited organelles of eukaryotic cells that are involved in numerous essential cell functions, e.g., energy metabolism, apoptotic pathways, and steroid hormone synthesis [1, 2]. The mitochondrial genome is optimized for functioning in women because the mitochondrial genome and mitogenome-nuclear genome interaction is effective in females only [3]. Recent studies report that female mitochondria generate half the amount of hydrogen peroxide compared to mitochondria of males and contain higher levels of antioxidant enzymes and compounds [4–9]. Female sex may be a risk factor for Alzheimer’s disease (AD), which may be explained by the longer life expectancy of women [10, 11]. However, women exhibit higher rates of AD than men even after adjusting for survival. Estimates from the *Aging, Demographics, and Memory Study* (ADAMS) revealed that 16% of women have Alzheimer’s disease or other dementias compared with 11% of men among people age 71 and older in the USA [12]. The prevalence of Alzheimer’s disease in Europe was estimated as 3.31% in men and 7.13% in women. The incidence of AD in Europe was 7.02 per 1000 person/years in men and 13.25 per 1000/years in women [13]. This disparity may be caused by differences in mitochondrial function between males and females. Sex-associated differences in the antioxidant capacity were examined [4–6, 14], and recent studies report that mitochondria in females of reproductive age generate half the amount of hydrogen peroxide [8]. Females also exhibit higher levels of antioxidant enzymes and compounds [4–9]. This sex difference decrease with age and following ovariectomy suggest a role of ovarian steroids [4, 6, 14, 15]. The decrease in gonadal hormone production during aging is gradual in men (testosterone), but estrogen levels in women promptly decrease after menopause [16, 17]. However, data of regarding sex-dependent differences in mitochondrial function, such as respiratory activity and oxidative phosphorylation, are rare.

Mitochondria-related reactive oxygen species (ROS), including hydrogen peroxide play a key role in neurodegenerative diseases, such as AD [8]. The brain of AD patients is marked by severe synapse and neuronal loss, atrophy, and depletion of neurotransmitter systems in the hippocampus and cerebral cortex [18, 19]. Recent findings suggest that these changes are induced by mitochondrial dysfunction (MD) and increased oxidative stress [20–24]. MD is detected at early stages of degenerative processes [24–26], and it represents a promising target for nutrient-based preventive strategies. Brain tissue from living humans is not accessible, and cerebral mitochondrial function cannot be directly studied in clinical investigations. However, animal studies demonstrated that mitochondrial function of peripheral thymocytes reflected mitochondrial function in brain cells [27]. Many observations of substantial mitochondrial dysfunction in human peripheral tissues, especially fibroblasts and blood cells (primarily platelets and lymphocytes) indicate a relation between peripheral and cerebral parameters, including age-related changes [28–32]. Increased oxidative stress during aging is not restricted to the brain; it is also present in peripheral cells, such as lymphocytes (a fractional part of peripheral mononuclear blood cells (PBMCs)). Leuner et al. observed mitochondrial dysfunction in peripheral blood cells isolated from Alzheimer’s patients that resulted in higher ROS production and oxidative-induced cell damage [32]. However, whether these changes reflect energy metabolism in the brain is not clear. We investigated sex-specific differences and correlated mitochondrial function in peripheral blood cells with brain energy metabolites in healthy participants to elucidate these issues. Mitochondrial function and adenosine triphosphate (ATP) levels were determined in isolated PBMCs, and concentrations of brain energy metabolites were measured using ^1^H-magnetic resonance spectroscopy (MRS) in the same individuals. MRS offers a non-invasive method for the measurement of brain metabolites, and its diagnostic potential for many neurological diseases as demonstrated in numerous studies, particularly cerebral amino acid *N*-acetylaspartate (NAA). NAA is highly enriched in neurons [33], and its concentration is correlated with neuronal density and damage. This metabolite is primarily synthesized in the mitochondria of neurons, and it is catalyzed by aspartate-*N*-acetyltransferase; moreover, the concentration of NAA corresponds to neuronal energy consumptions [17, 33, 34], which supports NAA as a marker of mitochondrial dysfunction in the brain [35–39].

The current pilot study examined possible sex-associated differences and correlated peripherally measured mitochondrial function with cerebral energy metabolites in females and males.

## Methods

### Study design and participants

Thirty healthy volunteers were recruited for the study. Six men dropped out because of blood sampling fears or withdrew their agreement for participation. Therefore, a convenience sample of 24 healthy volunteers (9 males/15 females, mean age 30.8 ± 7.1 years) was included in the cross-sectional study. The Ethics Committee of the Goethe University of Frankfurt, Germany (reference no. 31/16) approved the study design, which was performed in agreement with the Declaration of Helsinki (Version Fortaleza 2012). All subjects declared that they understood the experimental procedure and signed a written informed consent.

All participants underwent brain scans at the Brain Imaging Centre Frankfurt, Germany, for assessment of brain structures and metabolites. Blood samples were collected in EDTA/K_2_-coated Sarstedt Monovetten (#02.1333.001) from each person and transferred immediately to the Department of Pharmacology (Goethe University, Biozentrum, Niederursel, Frankfurt) for further analyses (e.g., determination of ATP levels and high-resolution respirometry). Weight, height, body mass index (BMI), waist-hip-ratio (WHR), and medical history were recorded (see Table 1). MRI exclusion criteria comprised cardiac pacemaker, neurostimulator, drug pump, metal parts in the body (metal clips, metal splinter), and claustrophobia. Exclusion criteria for blood sampling included hemophilia, hematophobia, or intake of anticoagulants.

**Table 1:** Demographic data

Participants	Male	Female
	9	15
Age [years]	30.2 ± 2.4	31.1 ± 1.8^ns^
Weight [kg]	86.0 ± 3.5	63.0 ± 3.2
Height [m]	1.82 ± 0.01	1.69 ± 0.01
Body mass index [kg/m^2^]	26 ± 1.1	22 ± 1.1
Waist-hip ratio	0.83 ± 0.03	0.78 ± 0.01
Smoker (*n*)	1	2

Values denote means ± SEM; ns = age differences were not significantly different (*p* = 0. 77)

### Isolation of peripheral blood mononuclear cells (PBMCs)

Peripheral blood mononuclear cells were isolated from fresh blood from healthy participants within 1–2 h after collection using density medium centrifugation and Ficoll-Paque PLUS (GE Healthcare Bio-Science, Darmstadt, Germany). Blood samples were collected either before or after MRS in EDTA/K2-coated Sarstedt Monovetten. Briefly, blood was diluted 1:1 with phosphate buffered saline (PBS), carefully layered onto Ficoll-Paque PLUS, and centrifuged at 400*g* for 40 min. Separated PBMCs were cautiously collected (2–4 ml), resuspended in 15 ml PBS, and centrifuged at 500*g* for 15 min. The supernatant was removed, and the pellet was resuspended in 15 ml PBS and centrifuged at 500*g* for 10 min [GE Healthcare info]. The supernatant was removed, and the pellet was resuspended in 1 ml RPMI Glutamax™-1 medium (11.1 mM glucose, supplemented with 3% FBS, 50 units/ml penicillin, 50 g/ml streptomycin; #61870) for ATP-measurement or 1 ml MIRO 5 for high-resolution respirometry (see below).

### Determination of ATP-levels in PBMC

To measure ATP concentrations, isolated PBMCs were resuspended in 1 ml RPMI medium, cultured in 96-well plate at a density of 1 × 10^5^ cells/100 μl/per well and incubated for 3 h in humidified atmosphere supplemented with 5% CO_2_ at 37 °C.

ATP levels were assessed after 3 h using the ViaLight®Plus bioluminescence kit (Lonza, Walkersville, USA), which is based on the production of light from ATP and Luciferin in the presence of the enzyme luciferase. A detailed description of the method was previously published [16]. The emitted light (bioluminescence) is linearly related to ATP concentration, and it was recorded using a luminometer (Victor21420 multilabel counter, Perkin Elmer, Rodgau-Jügesheim, Germany) [16]. The ATP concentration was normalized to cell number.

### High-resolution respirometry in permeabilized PBMCs

For the high-resolution respirometry, isolated PBMCs were resuspended in 1 ml MiR05, which is a mitochondrial respiration medium developed by Oroboros [40] containing EGTA (0.5 mM), magnesium dichloride (3 mM), lactobionic acid (60 mM), taurine (20 mM), potassium dihydrogenphosphate (10 mM), HEPES (20 mM), sucrose (110 mM), and essential fatty acid free bovine serum albumin (1 g/l). Cell density was adjusted to 10^−6^ cells per ml. An Oxygraph-2k system (Oroboros Instruments, Innsbruck, Austria) and the DatLab software version 4.3.2.7 were used to analyze mitochondrial respiration.

A complex protocol (elaborated by Prof. Dr. Erich Gnaiger, University of Innsbruck, Austria) was used to investigate the function of the respiratory system, including different substrates, uncouplers, and inhibitors. The cell suspension (2 ml) was added to the two chambers of the Oxygraph-2k, and the chambers were closed to stabilize respiration (endogenous respiration). The plasma membrane of the cells was permeabilized with digitonin (1 μg/10^−6^ cells), which leaves the mitochondrial outer and inner membranes intact. The capacity of oxidative phosphorylation was determined using complex I-related substrates (CI) glutamate (10 mM), malate (2 mM), and ADP (2 mM) followed by the addition of succinate (10 mM; OXPHOS). Leak respiration after the addition of glutamate/malate was labeled leak (G/M) and corresponded to state 4 respiration. Further addition of ADP induced state 3 respiration. The addition of oligomycin (2 μg/ml) allowed measurement of the state 2 respiration leak (omy). Uncoupling (ETS) was achieved with addition of carbonyl cyanide p-(trifluoromethoxy) phenylhydrazone (FCCP), injected stepwise up to 2.5 μM), and complex II respiration in the non-coupled state (CII_ETS_) was monitored after the addition of rotenone (0.5 μM) to the chambers. Residual oxygen consumption (ROX), which is oxygen consumption caused by enzymes outside the electron transfer system, was determined after inhibition of complex III via the addition of antimycin A (2.5 μM) and was subtracted from all respiratory parameters. COX activity (CIV) was measured after ROX determination and application of 0.5 mM tetramethyl-phenylenediamine (TMPD) as an artificial substrate of complex IV and 2 mM ascorbate to maintain TMPD in the reduced state. The autoxidation rate was determined after the addition of sodium azide (≥ 100 mM), and COX respiration was corrected for autoxidation [16]. The data were normalized to citrate synthase (CS) activity [pmol/(s*IU CS)].

### Citrate synthase (CS) activity

A subsample of isolated PBMCs was immediately frozen in liquid nitrogen and stored at − 80 °C for photometric determination of citrate synthase activity. Measurements were performed in duplicate. A detailed description of the method was published previously [16]. CS activity was normalized to IU per 1 × 10^6^ cells/ml.

### Protein quantification

The Pierce TM BCA Protein Assay Kit (Thermo Fisher Scientific, Waltham, MA, USA) was used to measure protein content. Bovine serum albumin was used as the standard.

### MR protocol

**Fig. 1:**
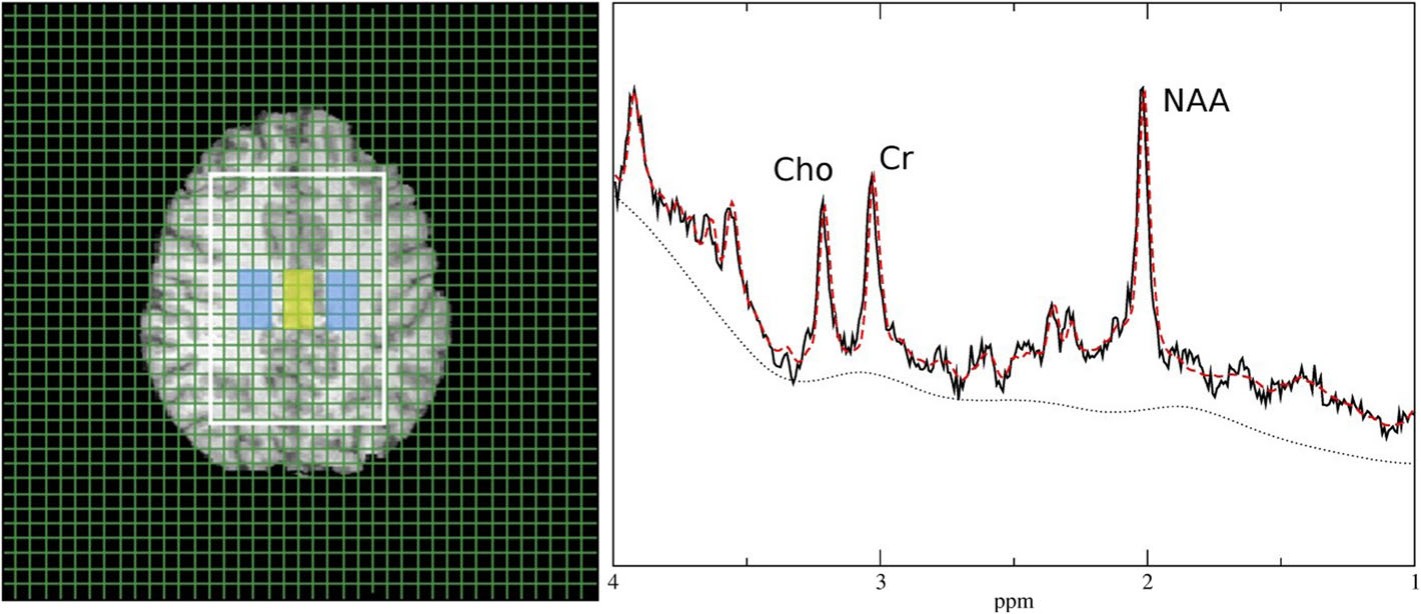
Target region and representative ^1^H spectrum from a GM voxel of the target yellow marked target region. Positions of the choline (Cho), creatine (Cr), and *N*-acetylaspartate (NAA) are marked in the spectrum. The red (broken) line shows the results of the LC mode-fitting procedure. The dotted line is the baseline as estimated by the LC Model. GM = gray matter

MRS of the brain was performed using a 3T whole body system (Magnetom Trio, Siemens Medical AG, Erlangen, Germany) equipped with a double tuned ^1^H/^31^P volume head coil (Rapid Biomedical, Rimpar, Germany). The protocol was similar to a previously published study protocol [41, 42] including ^1^H and ^31^P magnetic resonance spectroscopic imaging (MRSI) examinations. An axial slice at the level of the centrum semiovale partially including the trunk of the corpus callosum was recorded for ^1^H spectroscopy using 2D MRSI and an acquisition-weighted circular phase encoding scheme on a 20 × 20 matrix, field-of-view (FOV) of 240 × 240 mm^2^, 12-mm slice thickness, nominal voxel size of 12 × 12 × 12 mm^3^, TR 1500 ms, TE 30 ms, and two acquisitions. The volume of interest (VOI) was selected using a combination of point-resolved spectroscopy (PRESS) and outer volume suppression, and it was adjusted to contain gray matter (GM) and white matter (WM) (Fig. 1). The matrix was extrapolated to 40 × 40 prior to Fourier transformation to produce a 6.0 × 6.0 mm^2^ in-plane grid size (Fig. 1).

We also obtained a B1 map [43] and an a 2D ^1^H MRSI data set recording of the free induction decay (FID) signal of unsuppressed water (25-mm slice thickness, FOV 240 mm^2^, matrix size 16 × 16 extrapolated to 32 × 32, delay before data acquisition 2.4 ms, 2° excitation pulse flip angle) for absolute quantification of metabolite concentrations. The slice was aligned to the water suppressed PRESS MRSI slice. A T1-weighted MRI data set was recorded for tissue segmentation.

### MR data processing

T1-weighted MRI data were segmented using the FAST tool in the FMRIB Software Library (FSL) toolbox [44]. B1 maps were calculated as described in Volz et al. 2010 [43] and registered to the T1-weighted data for each subject. Parameter maps (GM, WM, B1) were registered to the spectroscopic data.

The ^1^H data spectra were fitted in the frequency domain using a linear combination of a set of model spectra including the main metabolites Cho (choline-containing compounds), Cr (creatine/phosphocreatine), and NAA (*N*-acetyl-aspartate and *N*-acetyl-aspartate-glutamate), using the software tool LCModel (Version 6.3, http://s-provencher.com). Metabolite signal intensities were corrected for T1 and T2 relaxation assuming previously published relaxation times at 3 T [45]. B1 inhomogeneity was taken into account, and metabolite concentrations of tissue water were calculated as described in the supplemental information (Additional file 1) [45].

A set of voxels from right and left WM and central GM were selected for further data evaluation (Fig. 1).

The ^31^P data were not included in this study.

### Chemicals

Chemicals were of the highest available purity and purchased from Sigma (St Louis, MO, USA) or Merck (Darmstadt, Germany) unless otherwise stated. Aqueous solutions were prepared using deionized, filtered water (Millipore, Billerica, MA, USA).

### Statistics

Values are presented as the means ± standard error of the mean (SEM), unless otherwise stated. Group differences were calculated using unpaired *t* test with Welch’s correction. Effect sizes were calculated using eta squared (*η*^2^) (Prism 7.03, GraphPad Software, San Diego, CA, USA). Statistical significance was defined for *p* values of *p < 0.05 and **p < 0.01.

## Results

### Sex-associated differences in peripheral mitochondrial parameters

Diversifications of mitochondrial efficacy and function are primarily dependent on alterations of the respiratory complex system. Mitochondrial respiration in isolated PBMCs was used as an effective method to assess mitochondrial efficacy and function in males and females.

**Fig. 2:**
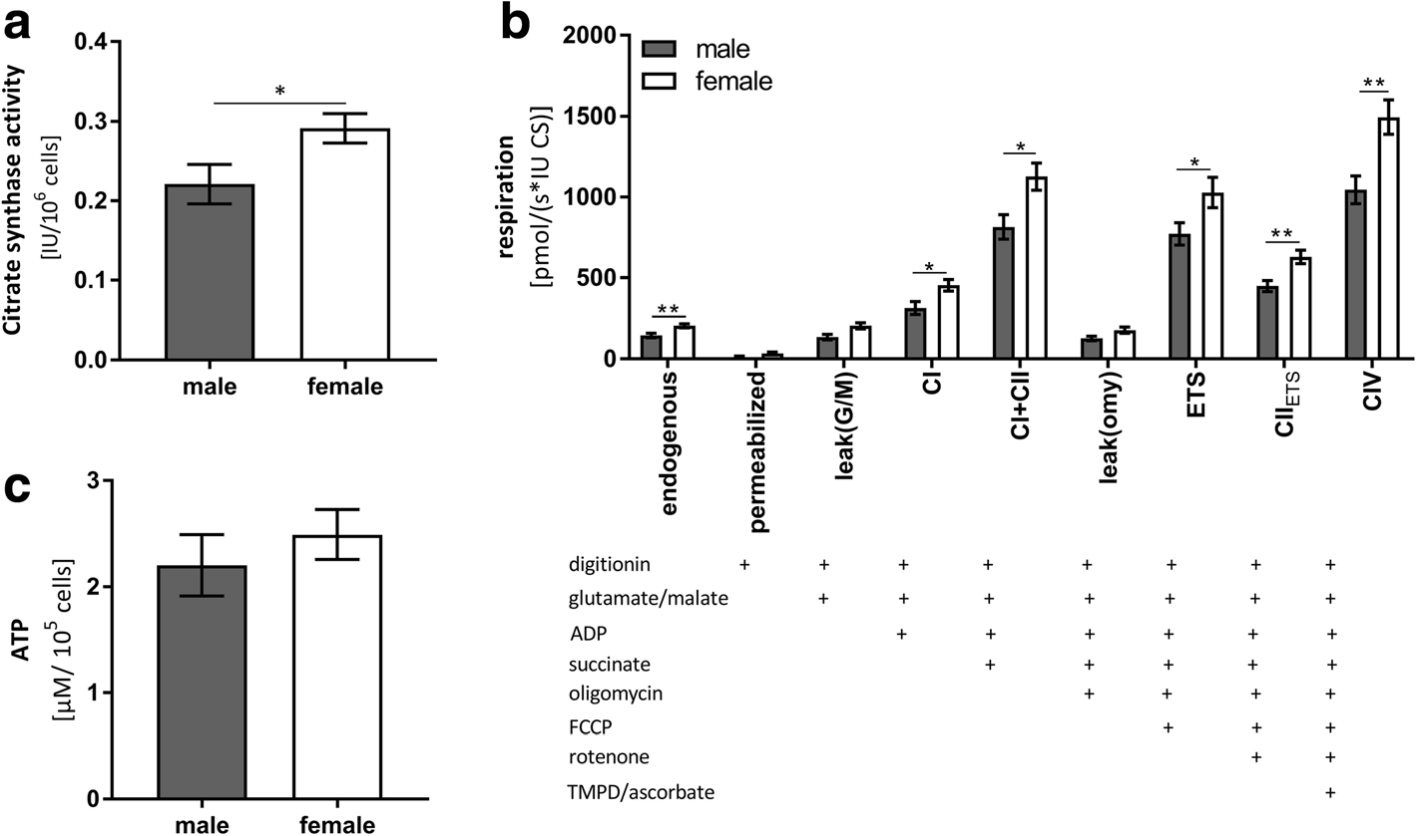
**a** Mitochondrial content marker CS activity was significantly higher in isolated PBMCs of females than those of males. Data represent means ± SEM; *n* = 21; unpaired *t* test with Welch’s correction; *η*^2^ = 0.29; *p* = 0.04; (*p < 0.05); CS = citrate synthase, PBMC = peripheral blood mononuclear cells. **b** Respiration of isolated PBMCs from healthy male and female participants in high-resolution respirometry. Values are normalized to international units (IU) of citrate synthase activity**.** The addition of a substance or inhibitor into the oxygraph chamber is indicated with a cross. Data represent means ± SEM; *n* = 19; unpaired *t* test with Welch’s correction were calculated for every state; *p* values and effect power are shown in Table 2; (*p < 0.05; **p < 0.01); PBMCs = peripheral blood mononuclear cells. **c** Cell count normalized ATP levels in isolated PBMCs of female and male participants. Data represent means ± SEM; *n* = 24; unpaired *t* test with Welch’s correction; *η*^2^ = 0.04; *p* = 0.4; PBMCs = peripheral blood mononuclear cells

CS is an enzyme of the Krebs cycle, and it is located in the mitochondrial matrix. CS activity is a mitochondrial mass marker [46]. CS activity was significantly higher in females compared to males (see Fig. 2a).

Mitochondrial complexes I, I+II, and IV and uncoupled respiration and ETS capacity in PBMCs isolated from males were significantly lower compared to females (see Fig. 2b). Mitochondrial respiration was normalized to CS activity, and the results refer to maximal respiratory per mitochondrion [46].

The activity of the mitochondrial respiration chain complexes (CI–IV) creates a proton gradient at the inner mitochondrial membrane. The resulting membrane potential ultimately represents the driving force for complex V (CV; F_1_/F_0_-ATPase) to produce ATP. ATP levels of female participants were approximately 10% higher compared to males (see Table 2) (see Fig. 2c). These differences were not significant, but the observed trends reflect lower mitochondrial respiration in PBMCs isolated from male volunteers (Fig. 2c).

**Table 2:** Measures of peripheral mitochondrial function and cerebral metabolism

	Women *n* = 15	Men *n* = 9	*p* value	*η* ^2^
O2k-Oxygraph [pmol/(s*IU CS)]				
Endogenous	205.8 ± 12.4	145 ± 14.8	0.007**	0.40
CI	453.6 ± 36.0	313 ± 39.6	0.02*	0.30
CI+II	1096.0 ± 86.4	814.5 ± 76.1	0.03*	0.27
ETS	1028 ± 94.8	772.2 ± 69.1	0.04*	0.22
CII_ETS_	612.8 ± 42.3	448.5 ± 34.5	0.008**	0.36
CIV	1495 ± 104.6	1045 ± 85.12	0.004**	0.40
CS activity [IU/10^6^ cells]	0.2914 ± 0.019	0.2211 ± 0.025	0.04*	0.29
ATP levels [μM/10^5^ cells]	2.50 ± 0.2	2.20 ± 0.3	0.45	0.04
NAA in GM [mmol/l]	12.27 ± 0.19	11.35 ± 0.25	0.009**	0.35
NAA in WM [mmol/l]	14.79 ± 0.38	13.32 ± 0.40	0.01*	0.25

Mean ± SEM, unpaired *t* test with Welch’s correction, significant differences are marked by asterisk, (*p<0.05; **p<0.01)

*ATP* adenosine triphosphate, *CS* citrate synthase, *C* complex, *ETS* electron transport system, *GM* gray matter, *NAA N*-acetylaspartate, *WM* white matter

#### Sex-associated differences in cerebral parameters

**Fig. 3:**
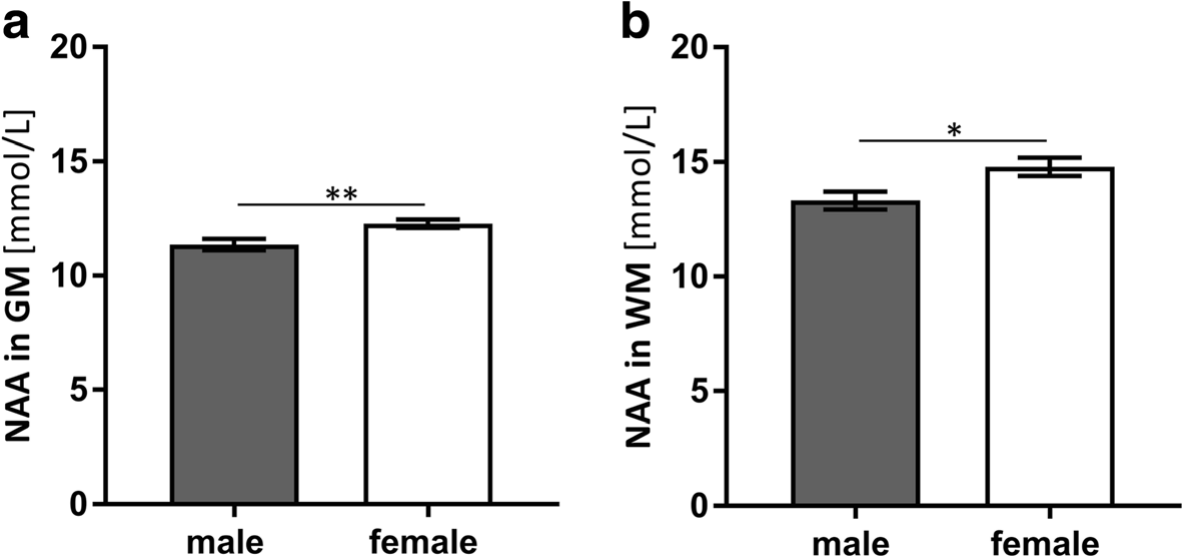
NAA concentrations in the **a** GM and **b** WM regions of male and female subjects. Concentrations are given in millimoles per tissue water volume. Female subjects show a significantly higher concentration. Data represent means ± SEM; *n* = 24; unpaired *t* test with Welch’s correction; **a** *η*^2^ = 0.35; *p* = 0.009; **b** *η*^2^ = 0.25; *p* = 0.01 (*p < 0.05; **p < 0.01); GM = gray matter, WM = white matter

NAA is a marker of neuronal energy consumptions [17, 33, 34]. Figure 3 shows the results from ^1^H-MR spectroscopic data from the brain. The NAA concentration was significantly higher in female participants compared to male participants in the total WM region and GM region.

### Correlation between mitochondrial function in PBMCs and the brain

**Fig. 4:**
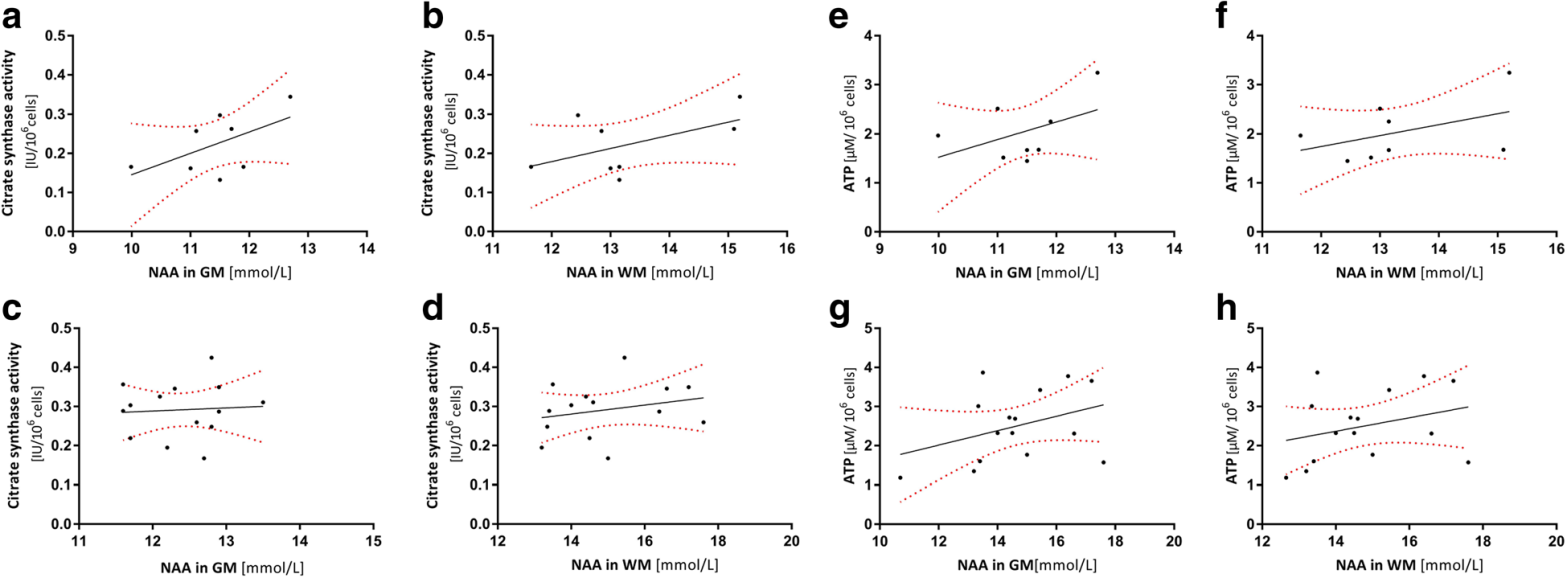
Correlation between citrate synthase (CS) activity and *N*-acetylaspartate (NAA) concentration of males in **a** gray matter (GM) and **b** white matter (WM) and females in **c** GM and **d** WM. Correlation between ATP levels and NAA concentrations of males in **e** GM and **f** WM and females in **g** GM and **h** WM. *n* = 22–24; *p* values and Pearson *r* are shown in Table 3

Individual content of NAA in GM and WM did not correlate with the respective ATP levels or citrate synthase activity in PBMCs (see Table 3 and Fig. 4).

**Table 3:** Correlations

Sex	Correlated values	Pearson r	*p* value
Male *n* = 9	NAA GM [mmol] vs. CS [IU/10^6^ cells]	0.55	0.15
	NAA WM [mmol] vs. CS [IU/10^6^ cells]	0.53	0.17
	NAA GM [mmol] vs. ATP levels [μM/10^5^ cells]	0.46	0.26
	NAA WM [mmol] vs. ATP levels [μM/10^5^ cells]	0.45	0.26
	NAA GM [mmol] vs. CI activity [pmol/(s*IU CS)]	0.34	0.33
	NAA WM [mmol] vs. CI activity [pmol/(s*IU CS)]	0.41	0.32
Female *n* = 15	NAA GM [mmol] vs. CS [IU/10^6^ cells]	0.06	0.82
	NAA WM [mmol] vs. CS [IU/10^6^ cells]	0.25	0.40
	NAA GM [mmol] vs. ATP levels [μM/10^5^ cells]	0.37	0.17
	NAA WM [mmol] vs. ATP levels [μM/10^5^ cells]	0.45	0.28
	NAA GM [mmol] vs. CI activity [pmol/(s*IU CS)]	0.30	0.38
	NAA WM [mmol] vs. CI activity [pmol/(s*IU CS)]	0.04	0.90

*ATP* adenosine triphosphate, *CI* complex I, *CS* citrate synthase, *GM* gray matter, *NAA N*-acetylaspartate, *WM* white matter

## Discussion

The present explorative pilot study measured peripheral markers of energy metabolism and compared these levels to the cerebral energy metabolite NAA. We observed sex-associated differences in mitochondrial function, ATP levels and citrate synthase activity in isolated PBMCs, and a significant effect of sex on the brain metabolite NAA.

### Sex-associated differences in cerebral and peripheral markers of energy metabolism

Mitochondrial complexes I, I+II, and IV and uncoupled respiration and ETS capacity in PBMC isolated from females were significantly higher compared to males (see Fig. 2b).

Activity of mitochondrial respiratory complexes CI–CIV is the driving force for ATP generation by complex V of the respiration chain. Therefore, the observed impairment in complex activities may account for the lower apparent ATP levels in males (see Fig. 2c). However, ATP levels were not significantly different in PBMCs isolated from men and women. This result may indicate that all participants were adequately supplied with cellular energy at the time of measurement. Notably, the respiration measurements determined the maximum possible oxygen consumption of the single respiratory complexes and thus, ATP levels may be much lower when damage (e.g., oxidative stress) occurs. CS activity was significantly higher in females compared to males (see Fig. 2a). These findings indicated that mitochondrial content is higher in females because CS activity, an enzyme of the mitochondrial Krebs cycle, is strongly associated with mitochondrial content [46]. Gaignard et al. detected no difference in CS activity between intact male and female rats, which emphasizes the importance of measuring this parameter in humans [6].

Our study found lower ATP levels and reduced mitochondrial function in PBMCs of males, which may indicate a generally lower mitochondrial function compared to women. This difference could produce the lower NAA concentrations in the brain. Mitochondria are the main source of NAA. Therefore, the significantly higher mitochondrial content may lead to significantly higher NAA concentrations in female brains. Maudsley et al. also found significant sex-associated differences in NAA levels in some brain regions of 41 male and 47 female participants (mean age = 33 years) [47]. The sex differences indicate increased NAA concentrations in brains of females relative to males, with an average of 4% in gray matter (GM) and white matter (WM).

Sex differences in mitochondrial function were measured in other tissues.

Rutkai et al. observed higher mitochondrial respiration in freshly harvested cerebral arteries from adult female rats compared to males [48].

Genetic differences in females and males may contribute to the observed sex differences in energy metabolism. Basic sex differences in mitochondrial metabolic regulation may exist because of the maternal inheritance of mitochondria. Mitochondria from females exhibit better coping with stressful conditions and are relatively resilient to DNA damage and mutations, which reduces the probability of producing inheritable metabolic disorders [7].

Sex steroids are another factor that may be responsible for the observed sex differences. The influence of sex hormones would also explain why women are more likely to develop Alzheimer’s disease than men, despite exhibiting better mitochondrial function (see Figs. 2 and 3), lower levels of ROS [8], and better antioxidant capacity [4–9] at a reproductive age. The decrease in female sex hormones after menopause reverses sex differences. Previous studies demonstrated the reduced antioxidant capacity and the increased ROS production after menopause [5, 6]. Comparison of mitochondrial function in pre- and postmenopausal women and men of the same age should be compared in a future study to confirm this mitochondrial respiration hypothesis. Pharmacological studies in young adult female rodents demonstrated that steroids influenced brain function. Ovariectomy decreased brain mitochondrial oxidative phosphorylation and increased oxidative stress [49–53]. Gaignard et al. demonstrated a sex difference in brain mitochondrial respiration and oxidative stress that is suppressed with aging and ovariectomy. These findings in reproductive animals are consistent with our results.

A direct genomic effect of estradiol is unlikely, because neither the antioxidant enzyme superoxide dismutase (SOD) nor glutathione peroxidase (GPx) contain estrogen-responsive elements in their promotor region. However, estradiol may activate mitogen-activated protein kinase (MAPK). MAPK activates the transcription factor NFκB, which upregulates the gene expression of antioxidant enzymes [54]. More antioxidant enzymes could protect the respiratory chain complexes against damage from ROS and explain the better mitochondrial respiration in females in reproductive age. Grimm et al. reviewed several studies that demonstrated an estrogen-induced upregulation of genes encoding for components of the mitochondrial electron transport chain, including CI, CIV, and the F1 subunit of ATP synthase, which is consistent with our detected significant differences in CI and CIV [15].

However, whether sex- or neurosteroids contributed to the differences in mitochondrial function in our study requires further investigation. The influence of the decrease in estradiol during menopause on the mitochondrial respiration should also be investigated. These findings emphasize the necessity to include males and females in experimental studies and particularly prevention strategies that target mitochondria.

#### Correlation between mitochondrial function in PBMCs and the brain

The higher level of peripheral markers of energy metabolism in female subjects was paralleled by a higher concentration of the metabolite NAA. Sex-associated differences were found in PBMCs and the brain, but statistical analyses revealed that the individual content of NAA in GM and WM did not correlate with the respective levels of ATP or citrate synthase activity in PBMCs (see Table 3). However, the effect size indicated a strong influence of the factor of sex on these parameters. The lack of significance may be due to the small number of subjects. Therefore, the lack of correlation does not necessarily indicate that energy metabolism in the brain and peripheral blood cells are not connected, especially because a tendency for positive correlations is observed on the graphs (Fig. 3). The lack of controls for other confounding variables, such as blood sampling times, smoking, and diet, is a further limitation of the study. Some recruited participants also dropped out, which resulted in an unequal number of women and men as another limitation of the study.

Several studies demonstrated mitochondrial dysfunction and increased ROS levels and apoptosis in lymphocytes of AD patients. Markers of mitochondrial function, such as NAA, also exhibited changes in the brains of AD patients compared to a control group. Mitochondrial function compromised in the peripheral cells and brains of AD patients, which is reflected in the altered *N*-acteylaspartate levels. *N*-acetylaspartate has been proposed as a marker of neuronal health, viability, and number [55]. Many ^1^H-MRS studies demonstrated decreased NAA concentration in dementia and other neurological disorders [56–61] where the NAA reduction is frequently located in regions of gray matter volume reduction. This reduction may indicate reduced neurons [62, 63] because NAA is highly concentrated in neurons. However, low NAA levels may reflect mitochondrial dysfunction because NAA concentration corresponds to mitochondrial function [33, 35–39, 62–64].

To our knowledge, this study is the first report to directly compare markers of mitochondrial function in the brain and PBMCs. Bartolotti et al. 2016 investigated the CAMP response element-binding protein (CREB) protein, which is important for the formation of memories [65]. CREB signaling is dysfunctional in mouse models of AD. These authors compared CREB expression in PBMCs and postmortem brain tissue. pCREB expression in PBMCs was positively correlated with pCREB expression in the postmortem PFC, which indicates that pCREB expression in PBMCs may reflect pCREB expression in the AD brain. Their results demonstrated impaired pCREB in AD brain and PBMCs, which reinforces our hypothesis of a connection between the changes in the brain and peripheral tissues, such as PBMCs during aging and pathology. These data confirms that AD is not a pure brain disease and emphasizes that mitochondrial function in PBMCs may be a feasible surrogate marker to detect differences in mitochondrial function in AD.

## Conclusion

Our study revealed sex-associated differences in mitochondrial function in healthy adults. Females exhibited significantly higher mitochondrial function in PBMCs than males. The mitochondrial brain metabolite NAA was also significantly higher in females compared to males. No significant correlation between individual parameters in PBMCs and brain were found, but our study suggests that mitochondrial function in PBMCs is a feasible surrogate marker to detect differences in mitochondrial function. Future studies should confirm our findings using a larger sample size and investigate the sex-associated differences in males and females older than 65 years compared to young women and men. Furthermore, we would extend the range of methods to determination of mtDNA copy numbers to further strengthen the sex-specific data.

## Additional file

Additional file 1: Calculation of B1 and coil-receive-profile-corrected metabolite concentrations normalized to tissue water. (PDF 58 kb)

## Abbreviations

1H: Proton
3 T: 3 Tesla
31P: 31-Phosphor
AD: Alzheimer’s disease
ADAMS: Aging, Demographics, and Memory Study
ATP: Adenosine triphosphate
BMI: Body mass index
C: Complex
Ch: Choline-containing compounds
Cre: Creatine/phosphocreatine
CREB: CAMP response element-binding protein
CS: Citrate synthase
DNA: Deoxyribonucleic acid
ETS: Electron transport system
FCCP: Carbonyl cyanide p-(trifluoromethoxy) phenylhydrazone
FID: Free induction decay
FOV: Field-of-view
FSL: FMRIB Software Library
GM: Gray matter
GPx: Glutathione peroxidase
IU: International unit
MAPK: Mitogen-activated protein kinase
MD: Mitochondrial dysfunction
MiR05: Mitochondrial respiration media 05
MRS: Magnetic resonance spectroscopy
MRSI: Magnetic resonance spectroscopic imaging
mtDNA: Mitochondrial DNA
NAA: *N*-Acetylaspartate
NFκB: Nuclear factor “kappa-light-chain-enhancer” of activated B cells
OXPHOS: Oxidative phosphorylation
PBMCs: Peripheral blood mononuclear cells
PBS: Phosphate buffered saline
pCREB: Phosphorylated cAMP response element-binding protein
PRESS: Point-resolved spectroscopy
ROS: Reactive oxygen species
ROX: Residual oxygen consumption
RPMI: Roswell Park Memorial Institute
SEM: Standard error of the mean
SOD: Superoxide dismutase
TMPD: Tetramethyl-phenylenediamine
VOI: Volume of interest
WHR: Waist-hip-ratio
WM: White matter
*η*^2^: Eta squared
