# Supplementary material for: Sex-associated differences in mitochondrial function in human peripheral blood mononuclear cells (PBMCs) and brain
Source: Biol Sex Differ. 2018 Jul 25;9:34. doi: 10.1186/s13293-018-0193-7 (PMC6060503; doi:10.1186/s13293-018-0193-7)
Supplement: Supplementary file 1 — Calculation of B1 and coil-receive-profile-corrected metabolite concentrations normalized to tissue water. (PDF 58 kb) [file 13293_2018_193_MOESM1_ESM.pdf]

Calculation of B1 and Coil-Receive-Profile corrected metabolite concentrations normalized to tissue water.

Metabolite signal intensities were corrected for T1 and T2 relaxation assuming previously published relaxation times at 3 T [44]. Further, B1 inhomogeneity were taken into account by division with $\sin^5(\alpha)$, where α, the real flip angle at the position of the voxel [45] , was calculated from the B1 maps. A receive profile of the coil RP_{coil} (including other constants related to signal amplification and postprocessing) can be determined by

$$RP_{Coil} = \frac{S}{C \cdot V_{mrsi}} \quad (1)$$

where S represents the measured signal of a substance at concentration C following a 90° pulse and V_{mrsi} the MRSI voxel size. RP_{coil} can be represented by a matrix with the dimension of the spectroscopic image. The metabolite concentration C_{met} can be calculated by

$$C_{met} = \frac{S_{met}}{\sin^5 \alpha \cdot RP_{coil} \cdot V_{mrsi}} \quad (2)$$

with S_{met} representing the relaxation corrected metabolite signals.

RP_{coil} at the slice of the ^{1}H MRSI data can be obtained from the FID MRSI data of water with

$$RP_{Coil} = \frac{S_{wat}}{\sin(0.22\alpha) \cdot C_{wat} \cdot V_{mrsi}} \quad (3)$$

S_{wat} represents the relaxation corrected water intensity while the term sin(0.022α) takes into account the effect of B1 inhomogeneity and the 2° flip angle. Due to the small flip angle and the short delay between excitation and acquisition, no relaxation corrections are required for water. The concentration C_{wat} in the voxel can be estimated with:

$$C_{wat} = (0.73\, GM_{fraction} + 0.64\, WM_{fraction} + CSF_{fraction}) \cdot 55\, mol/l \quad (4)$$

assuming 73% water in the GM fraction ($GM_{fraction}$), 64% water in the WM fraction ($WM_{fraction}$) and 100 % water in the CSF fraction ($CSF_{fraction}$). The RP_{coil} matrix obtained for water was adjusted to to the matrix for PRESS CSI by interpolation and then used in equation (2) to calculate C_{met}. Finally, the absolute concentrations referred to tissue water (C_{metwat}) were calculated by

$$C_{metwat} = \frac{C_{met}}{GM_{fraction} + WM_{fraction}} \quad (5)$$
